# Big Data Analytics and Structural Health Monitoring: A Statistical Pattern Recognition-Based Approach

**DOI:** 10.3390/s20082328

**Published:** 2020-04-19

**Authors:** Alireza Entezami, Hassan Sarmadi, Behshid Behkamal, Stefano Mariani

**Affiliations:** 1 Department of Civil and Environmental Engineering, Politecnico di Milano, 20133 Milano, Italy; stefano.mariani@polimi.it; 2 Department of Civil Engineering, Faculty of Engineering, Ferdowsi University of Mashhad, Mashhad 9177948944, Iran; hassan.sarmadi@mail.um.ac.ir; 3 Department of Computer Engineering, Faculty of Engineering, Ferdowsi University of Mashhad, Mashhad 9177948944, Iran; behkamal@um.ac.ir

**Keywords:** structural health monitoring, big data, statistical pattern recognition, time series analysis, Kullback–Leibler divergence, nearest neighbor, large-scale bridges

## Abstract

Recent advances in sensor technologies and data acquisition systems opened up the era of big data in the field of structural health monitoring (SHM). Data-driven methods based on statistical pattern recognition provide outstanding opportunities to implement a long-term SHM strategy, by exploiting measured vibration data. However, their main limitation, due to big data or high-dimensional features, is linked to the complex and time-consuming procedures for feature extraction and/or statistical decision-making. To cope with this issue, in this article we propose a strategy based on autoregressive moving average (ARMA) modeling for feature extraction, and on an innovative hybrid divergence-based method for feature classification. Data relevant to a cable-stayed bridge are accounted for to assess the effectiveness and efficiency of the proposed method. The results show that the offered hybrid divergence-based method, in conjunction with ARMA modeling, succeeds in detecting damage in cases strongly characterized by big data.

## 1. Introduction

Civil structures are currently facing issues related to aging, material deterioration, excessive loading conditions unexpected at the design stage, inappropriate usage, environmental actions and natural hazards. Under such circumstances, they may get affected by serious damages which threaten their structural performance and safety. To avoid irreparable events and guarantee the serviceability of the structures, structural health monitoring (SHM) represents a practical tool to evaluate the structural conditions both at global and local levels [1,2,3]. To achieve this objective, relatively dense sensor networks need to be designed and data acquisition must be exploited to continuously collect information in terms of, e.g., structural vibrations [4,5,6,7,8,9,10]. 

Recent advances in sensor and information technologies have opened up the possibility of exploiting big data, in order to shift the focus from sensing and instrumentation to the analysis and interpretation of sensor network outcomes via data-driven methods [11]. Big data is a term associated with a large volume of high-dimensional data, whose size is beyond the ability of commonly used software and hardware to analyze the samples in a limited amount of time [12]. The concept of big data has received remarkable attention when dealing with complex engineering problems, also within the civil engineering community [13,14,15]. Big data may arise for SHM in the case of long-term monitoring strategies, use of dense sensor networks, exploitation of multiple dynamic tests on the structure and high sampling rates [16].

Big data analytics for SHM is a relatively new research topic. In [16], challenges related to big data were discussed on the basis of their characteristics like variety (type and nature of data coming from different sources), volume (size and quantity of stored data), velocity (speed at which the data are processed) and complexity (related to uncertainties and inaccuracies in them). In [17], the computational sensitivity of common SHM procedures was assessed in relation to system identification and damage detection, in the case of large volumes of vibration measurements to be processed. A machine learning algorithm was proposed in [11], based on cross-correlation and robust regression analyses, for processing data collected from the mechanical components of movable bridges. A method was also offered in [18], based on the statistical pattern recognition paradigm to include the use of multivariate analysis, sensor data fusion and machine learning for damage detection from a large volume of data acquired from distributed piezoelectric sensors. Damage detection using distributed parallel processing was implemented in [12], with the aim of addressing the issues linked to the volume and variety of the data. Big data analytics were carried out in [19] for the condition evaluation of highway bridges, by roughly considering one million data samples obtained from the National Bridge Inventory. In [20], the focus was on structural damage detection and localization by handling big data through an iterative spatial compressive sensing algorithm.

The processing of data in long-term SHM may be a complex and time-consuming procedure, often preventing the monitoring system to work in real-time. Further to that, a large volume of high-dimensional data (e.g., the acceleration time histories acquired by a dense sensor network) needs a vast storage space, detrimentally affecting the performance of the software used for data analytics [21]. What precedes must also deal with issues related to uncertainties such as noise, environmental and operational variability due to temperature fluctuation, humidity variation and mass changes caused by traffic loads [18,22,23]. For a long-term SHM program, the measurement of vibrations takes place under different environmental and operational conditions, leading in some cases to changes in the structural response similar to those caused by damage, and hence providing false alarms [22]. 

Data-driven methods for SHM have been inspired by the theory of statistical pattern recognition [23,24,25,26,27]. These methods consist of two main steps: extraction of damage-sensitive features from periodically spaced vibration measurements over a period of time, and analysis of these features via statistical approaches, to assess the current state of the structure. The reason to move to damage-sensitive features lies in the fact that the direct use of raw vibration data may not be sufficiently informative [11]. As vibration data are acquired in time, time series analysis provides an efficient tool for feature extraction [28,29,30,31,32]. 

The analysis of the damage-sensitive features for damage detection is usually carried out via statistical techniques. In fact, the definition of a meaningful relationship between damage and the features extracted from the raw vibration data, on the basis of physical laws or numerical models of the structure, proves difficult if not impossible [25]. The analysis of damage-sensitive features is usually known as statistical decision-making or feature classification (see [18,22,23,29,33,34,35]). Within SHM, this process aims to compare the features relevant to two different structural conditions, one of which is assumed normal or undamaged, and then make a decision about the current state of the structure, which may be either undamaged or damaged. From a statistical viewpoint, distance metrics for feature classification have to provide a measure of the discrepancies between two sets of data samples, handled as random variables, in terms of, e.g., their probability distributions [36]. There exist effective univariate and multivariate distance metrics that can be adopted in SHM analysis [22,23,25,29,30,31,37,38]; however, their use does not always guarantee an accurate and reliable feature classification, particularly in the case of big data analytics.

Having considered the above-mentioned limitations, the main objective of this work is to propose a data-driven method for SHM based on statistical pattern recognition in the presence of big data. First, ARMA representations are adopted to model, in the time-domain, the vibration responses, which are assumed to consist of large volumes of high-dimensional data, and reliably extract damage-sensitive features in a low-dimensional space. Second, a hybrid divergence-based method is used to take a decision about damage occurrence. Such a method is a combination of a partition-based Kullback–Leibler divergence (PKLD) and the nearest neighbor (NN) rule, and is, therefore, termed PKLD-NN. It stands as an improvement over a classical hybrid method obtained by combining the Euclidean-squared distance (ESD) and the NN rule (ESD-NN), as proposed in [39]. Furthermore, the PKLD improves the conventional Kullback–Leibler divergence (KLD) in measuring the discrepancy between two sets of time series samples, to enable addressing the main limitations for random samples and coping with high-dimensional features for damage diagnosis. The high detectability of damage and the utility of long-term SHM methods are shown for the proposed PKLD-NN approach, accounting also for ambient vibrations and environmental and/or operational variability conditions. A major strength of the proposed approach is its capability to provide a novelty detection on the basis of the measured data and low-dimensional feature samples, independently of the specific type of damage. Experimental datasets of a large-scale cable-stayed bridge are adopted to verify the effectiveness and efficiency of the proposed data-driven method. Through comparison with state-of-the-art techniques, the newly proposed strategy is reported to be highly successful in detecting damage and handling big data. 

The remainder of this paper is organized as follows. Section 2 briefly addresses the vibration response modeling by a time series analysis for feature extraction. Section 3 introduces the hybrid divergence-based approaches for feature classification. The results of the feature extraction and feature classification techniques for the mentioned cable-stayed bridge are gathered in Section 4. Finally, Section 5 draws main conclusions of the present work and future prospects.

## 2. Feature Extraction by ARMA Modeling

Time series modeling is a powerful tool for feature extraction [28,29]. When the excitation (input) and the response (output) data are both available, feature extraction via time series modeling is an input–output problem; in such a case, the autoregressive with an exogenous input (ARX) models and autoregressive moving average with an exogenous input (ARMAX) models are the most suitable ones. When the excitation data is instead unmeasurable or unknown (like in the case of ambient vibrations), feature extraction is an output-only problem; in this case, the most proper time series models are the autoregressive (AR) and autoregressive moving average (ARMA) ones. The above-mentioned time series models generally consist of the output or AR, input or X, and error or MA terms [40]. From an engineering perspective, if the measured structural output is induced by ambient vibrations, time series models must cope with the MA or error term [28]; hence, the ARMA model represents the most appropriate representation for modeling these cases [28,31].

Modeling of the structural response via ARMA representations is a parametric method for time series-based feature extraction. By fitting an ARMA model to a measured vibration response, model orders and coefficients can be estimated, allowing next to extract some statistical characteristics as damage-sensitive features. ARMA models are also known to provide a more parsimonious representation than the AR ones (see, e.g., [28,41]).

Let *y*(*t*) be a measured vibration response of the structure at time *t,* caused by the ambient excitation. Provided that *y*(*t*) is linear and stationary, the ARMA model reads:

(1) $$y\left(t\right)=\sum_{i=1}^{p}\varphi_{i}y\left(t-i\right)+\sum_{j=1}^{q}\psi_{j}e\left(t-i\right)+e\left(t\right),$$

where the first and second sums at the right-hand side respectively refer to the AR (output) and MA (error) terms of the whole model. In Equation (1): *p* and *q* denote the two model orders; *φ*_1_…*φ_p_* and *ψ*_1_…*ψ_q_* are the coefficients of the AR and MA terms; and *e*(*t*) is the residual at time *t*, which represents the difference between the actually measured response and the one predicted by the model. A strength of this representation is that the coefficients of the AR term are directly related to the inherent physical properties of the structure [31]; moreover, the MA term is linked to the excitation source, so that any change in its amplitude results in a variation of the MA coefficients [31].

For the process of feature extraction, the AR coefficients and the residuals of the ARMA model can be adopted as the main damage-sensitive features. Table 1 gathers the steps of the resulting coefficient-based feature extraction (CBFE) and residual-based feature extraction (RBFE) algorithms based on ARMA modeling. In Step 1, the orders of the ARMA model are defined by any order determination method. Step 2 is intended to estimate the unknown coefficients of the AR and MA terms, through the prediction error technique. These initial two initial steps are common for the CBFE and RBFE algorithms. In Step 3, the AR coefficients and the ARMA residuals are extracted as damage-sensitive features of the undamaged condition for the CBFE and RBFE algorithms, respectively. It has to be mentioned that Steps 1–3 are carried out by using only the vibration data referring to the undamaged structural condition. For the next Steps 4–6, the vibration data relevant to the current state and the model information related to the undamaged state must be exploited. In Step 4, the already obtained orders are adopted for modeling the vibration response in the current state. For the CBFE algorithm, in Step 5a, the new AR and MA coefficients are estimated by the same prediction error technique adopted in Step 2; for the RBFE algorithm, the model coefficients already tuned in Step 2 are instead used. Finally, in Step 6a, the AR coefficients for the CBFE algorithm and in Step 6b, the ARMA residuals for the RBFE algorithm are extracted as damage-sensitive features for the current state.

## 3. Hybrid Divergence-Based Methods

For data-driven SHM strategies based on statistical pattern recognition, hybrid approaches were already proposed for sensing [42], feature extraction [30,31,43] and statistical decision-making [22,44,45]. In the following, details are provided for the classical ESD-NN and for the newly proposed PKLD-NN methods, both of which are suitable for statistical decision-making and feature classification.

The considered techniques are hybridized algorithms resting on the NN *f*-divergence estimator [39]. The ESD-NN technique exploits the traditional ESD to find the NN, whereas the PKLD-NN method is based on the use of the PKLD; it should be noted that ESD and PKLD are the distance and divergence measures, respectively. From a statistical viewpoint, the two strategies are similar as they look for dissimilarities between two sets (vectors) of data or feature samples. On the other hand, their main differences are related to the statistical properties; the distance measures such as ESD are metric and symmetric, and the divergence measures such as PKLD may be non-metric and non-symmetric. In spite of the applicability of statistical distance measures to any type of data, divergence measures are known to be more suitable to quantify the dissimilarities between two probability distributions [36].

### 3.1. ESD-NN

Let **X** = [**x**_1_…**x***_i_*…**x***_n_*] and **Z** = [**z**_1_…**z***_j_*…**z***_m_*] be two matrices respectively gathering the *n* and *m* feature vectors, each with *r* samples. Vectors **x***_i_,* with *i* = 1,…,*n*, and **z***_j_*, with *j* = 1,…,*m*, all include high-dimensional features as r≫n,m.

The goal of the ESD-NN technique is to find the NN of each feature vector of **X** and **Z** via the ESD. The NN of **x***_i_* in **x**_1_…**x***_i_*_−1_,**x***_i_*_+1_…**x***_n_* is found by means of

(2) $$E_{xx}\left(i\right)=\min\left(\sum_{k=1,k\neq i}^{n}\sum_{l=1}^{r}{\left(x_{il}-x_{kl}\right)}^{2}\right),$$

which denotes the smallest ESD value for **x***_i_*. Equation (2) thus provides a scalar value for *E_xx_*, equal to the minimum distance between the *r*-dimensional vectors **x***_i_* and **x***_k_*. After having considered all the feature vectors of **X**, the *n*-dimensional vector of the ESD values **E_xx_** = [*E_xx_*(*i*)… *E_xx_*(*n*)] is obtained. The same process is followed for the feature vectors of **Z**, in order to find the NN of **x***_i_* in **z**_1_…**z***_m_*. Accordingly, the ESD is given by

(3) $$E_{xz}\left(i\right)=\min\left(\sum_{j=1}^{m}\sum_{l=1}^{r}{\left(x_{il}-x_{jl}\right)}^{2}\right),$$

which is now the smallest ESD value relevant to **x***_i_* for all the feature vectors of **Z**. For the feature vectors of **X**, the *n*-dimensional vector of the ESD quantities is obtained as **E_xz_** = [*E_xz_*(*i*)… *E_xz_*(*n*)]. The discrepancy between **X** and **Z** is finally computed by handling the vectors **E_xx_** and **E_xz_** as follows:

(4) $$d_{EN}\left(i,k\right)=\frac{r}{n}\log\left(\frac{E_{xz}\left(i\right)}{E_{xx}\left(k\right)}\right)+\log\left(\frac{m}{n-1}\right),$$

where *k*,*i* = 1,…,*n*. By arranging all the entries of the matrix obtained here above with Equation (4) into a one-dimensional array, the *n*^2^-dimensional vector **d_EN_** = [*d_EN_*(1)… *d_EN_*(*n*^2^)] is assembled.

The same process is performed to compute the NN *f*-divergence values for the vector **E_xx_** alone, according to:

(5) $$d_{ET}\left(i,k\right)=\frac{r}{n}\log\left(\frac{E_{xx}\left(i\right)}{E_{xx}\left(k\right)}\right)+\log\left(\frac{n}{n-1}\right).$$

If all the entries of this matrix are arranged again into a one-dimensional array, to obtain the *n*^2^-dimensional vector **d_ET_** = [*d_ET_*(1)…*d_ET_*(*n*^2^)], this vector can be used to define the threshold limit for damage detection via a standard confidence interval method under an assigned significance level. The formerly introduced vectorization of the matrices obtained with Equations (4) and (5) facilitates the evaluation of the NN *f*-divergence values for early damage detection. Further to that, the values in vectors **d_ET_** and **d_EN_** are finally merged to define vector **d_N_** = [*d_ET_*(1)…*d_ET_*(*n*^2^)…*d_EN_*(1)…*d_EN_*(*n*^2^)], to ease the comparison of the normal and current states in the results section.

### 3.2. PKLD-NN

The proposed PKLD-NN method differs from the formerly discussed one as it provides a dissimilarity calculation between **X** and **Z** based on PKLD, in place of the conventional ESD. The PKLD is an *f*-divergence approach that can be exploited to measure the discrepancy between two vectors of random high-dimensional data. It is centered around a segmentation algorithm for random samples to subdivide them into independent segments, used next in the divergence calculation. Recently, in [23], the use of PKLD was proposed to locate damage on the basis of the AR model residuals handled as random high-dimensional damage-sensitive features; the approach has shown to be effective and efficient for SHM, even under environmental and operational variability conditions, when the damage-sensitive features are high-dimensional. Accordingly, if **X** and **Z** are two sets of high-dimensional feature samples with unpredictable uncertainties, the PKLD is expected to provide a better performance with respect to ESD. 

The dissimilarity calculation via PKLD is carried out by arranging each vector **z***_j_* in an ascending order. A segmentation algorithm is introduced next, based on maximum entropy [23], to divide the arranged vector **z***_j_* into *c* segments. The total number of segments thus introduced is given by *c* = *r*/*β*, where *β* = $\sqrt{r}$. As *β* and *c* should be positive integers, their values must be always rounded off to the nearest integer. With the exception of the last one, all the segments *z*_(*h*−1)*β*_ < **S***_h_* ≤ *z_hβ_*, with *h* = 2,…,*c*−1, have the same dimension; the last segment instead collects the entries *z*_(*c*−1)*β*_ < **S***_c_* ≤ *z_max_*. After the segmentation, PKLD measures the dissimilarity between **x***_i_* and **z***_j_* in the following way:

(6) $$d_{PKLD}\left(x_{i}||z_{j}\right)=\sum_{h=1}^{c-1}\frac{\alpha_{h}}{r}\cdot \left(\log\left(\frac{\alpha_{h}}{r}\right)-\log\left(\frac{\beta }{r}\right)\right)+\frac{\alpha_{c}}{r}\cdot \left(\log\left(\frac{\alpha_{c}}{r}\right)-\log\left(2\gamma_{c}\right)\right),$$

where *γ_c_* = (*r*−*βc*)/*r* is a correction factor for the last segment **S***_c_*; *α_h_* and *α_c_* are the numbers of samples of **x***_i_* which fall within the domain of the *h*^th^ and last segments of **z***_j_*, respectively. Note that the vector **x***_i_* does not need to be re-arranged in this procedure. 

The proposed PKLD-NN method follows the same procedure described for the ESD-NN technique. Hence, the NN of **x***_i_* in **x**_1_…**x***_i_*_−1_,**x***_i_*_+1_…**x***_n_* is provided by PKLD as follows:

(7) $$P_{xx}\left(i\right)=\min\left(\sum_{k=1,k\neq i}^{n}d_{PKLD}\left(x_{i}||x_{k}\right)\right).$$

and it is given by the smallest PKLD value for **x***_i_*. For all the feature vectors, the *n*-dimensional vector of the PKLD values finally reads **P_xx_** = [*P_xx_*(1)…*P_xx_*(*n*)]. The same approach is followed for the feature vectors of **Z**, to compute the NN of **x***_i_* in **z**_1_…**z***_m_* in the following form:

(8) $$P_{xz}\left(i\right)=\min\left(\sum_{j=1}^{m}d_{PKLD}\left(x_{i}||z_{j}\right)\right),$$

and the *n*-dimensional vector of the PKLD values is provided as **P_xz_** = [*P_xz_*(1)…*P_xz_*(*n*)]. Finally, the dissimilarity between **X** and **Z** is estimated via the NN *f*-divergence as in Equation (4), namely through

(9) $$d_{PN}\left(i,k\right)=\frac{r}{n}\log\left(\frac{P_{xz}\left(i\right)}{P_{xx}\left(k\right)}\right)+\log\left(\frac{m}{n-1}\right),$$

to define the *n*^2^-dimensional vector **d_PN_** = [*d_PN_*(1)… *d_PN_*(*n*^2^)]. By handling the vector **P_xx_** only, the divergence from the normal condition is next given by

(10) $$d_{PT}\left(i,k\right)=\frac{r}{n}\log\left(\frac{P_{xx}\left(i\right)}{P_{xx}\left(k\right)}\right)+\log\left(\frac{n}{n-1}\right),$$

to define the *n*^2^-dimensional vector **d_PT_** = [*d_PT_*(1)…*d_PT_*(*n*^2^)]. For early damage detection, the matrices obtained with Equations (9) and (10) are re-arranged into the vectors **d_PN_** and **d_PT_**. The entries in **d_PT_** are then used to compute a threshold limit as for the ESD-NN approach, and the values in vectors **d_PN_** and **d_PT_** are all gathered into **d_P_** = [*d_PT_*(1)…*d_PT_*(*n*^2^)…*d_PN_*(1)… *d_PN_*(*n*^2^)].

### 3.3. Decision-Making for SHM

The process of decision-making for SHM tries to assess the current state of the structure and to provide a decision concerning whether this state is affected by a damage or not. For this purpose, it is necessary to compare the NN divergence values with the threshold limit; if the feature matrix **Z** comes from a damaged state, any deviation of the entries in the vector **d_PN_** or **d_EN_** is representative of a damage occurrence. If the NN methods mistakenly classify the structure as undamaged, this state is termed false negative, or Type II false detection. On the other hand, since the feature matrix **X** belongs to the normal condition, all the divergence values in **d_PT_** or **d_ET_** are supposed not to exceed the threshold limit; if the methods incorrectly classify the undamaged state as damaged, a false positive, or Type I false alarm occurs.

## 4. Performance Evaluation and Verification

In this section, a series of large-scale experimental datasets relevant to the Tianjin Yonghe Bridge [46] are employed to evaluate the performance of the proposed data-driven SHM strategy and verify its accuracy and effectiveness. 

### 4.1. Bridge Description

**Figure 1:**
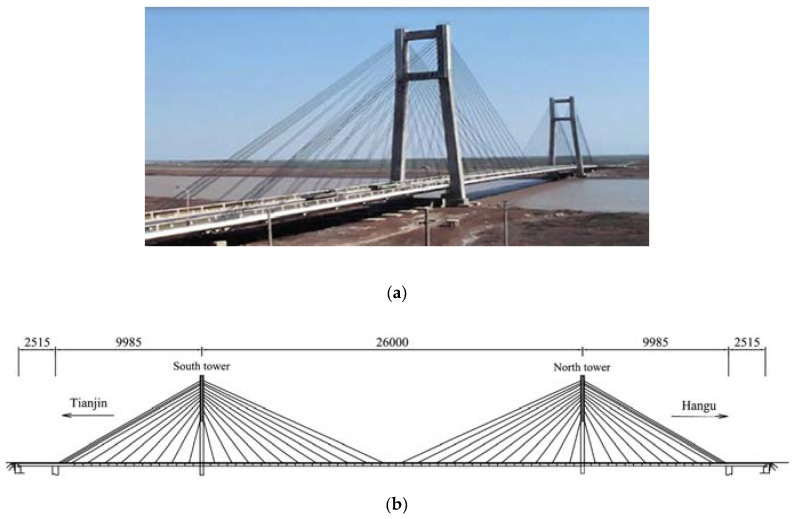
The Tianjin Yonghe Bridge: (**a**) general view, (**b**) main dimensions.

Figure 1 provides a general view and the main dimensions of the Tianjin Yonghe cable-stayed bridge, which is one of the earliest cable-stayed bridges constructed in mainland China. It is characterized by a main span of 260 m, and two side spans of 25.15 m and 99.85 m; overall, it is 510 m long and 11 m wide. The concrete towers, connected by two transverse beams, are 60.5 m tall.

The bridge was opened to traffic on December 1987. After 19 years of operation, in 2005 some cracks were found at the bottom of a girder segment over the mid-span; some stayed cables near the anchors were also recognized to be severely corroded. The Center of Structural Monitoring and Control (SMC) at the Harbin Institute of Technology equipped the bridge with a sophisticated SHM system, and monitored the data measured after a major rehabilitation program for replacing the damaged girder segment and all the stay cables. Acceleration time histories were acquired by 14 single-axis accelerometers during 12 days, from January to August 2008. During a routine inspection of the bridge in August 2008, new damage patterns were found in the girders.

The data collected each day consist of 24 sets of one-hour measurements, with a sampling frequency of 100 Hz. The acceleration response at each sensor location thus consists of 360,000 data samples. As the sensor #10 did not provide meaningful measurements, in this study the acceleration responses from the other 13 sensors have been considered, as acquired on January 1, January 17, February 3, March 19, March 30, April 19, May 5, May 18 and July 31. The measurements gathered on the first eight days have been associated with the normal conditions of the bridge, whereas the last day only refers to the damaged state.

Taking into account the measured acceleration responses along the 24 test measurements, the data samples for feature extraction by ARMA modeling and statistical decision-making through the proposed PKLD-NN method totally amount to 1,010,880,000, then to 112,320,000 samples for each day of measurements. Such samples represent a huge volume of high-dimensional data, that for instance occupy by themselves 2.59 GB of hard disk drive (HDD) space.

### 4.2. Feature Extraction

Signal pre-processing has been first carried out for data standardization and detrending. The CBFE and RBFE algorithms have been respectively adopted to extract the AR coefficients and the residuals of the ARMA model, as damage-sensitive features. According to Table 1, the first step of both algorithms is the determination of the orders of the ARMA model with the vibration data of the undamaged state. The iterative order determination technique proposed in [29] has been adopted to obtain the values of *p* and *q* (see Equation (1)) for each acceleration response representative of the normal conditions. Such a technique is based on the correlation analysis of the residual samples by the Ljung–Box Q-test (LBQ test) [40], and is able to set the aforementioned orders of time-invariant linear models. By enforcing *p* = *q* to speed up the order determination stage of the procedure, the iterative algorithm defines the orders so that the residuals of the model become uncorrelated [29]. This process has been carried out for all the vibration responses of the 13 sensors in the 24 test measurements. Therefore, 192 = 24 × 8 orders, possibly different, have been obtained at each sensor location. 

**Figure 2:**
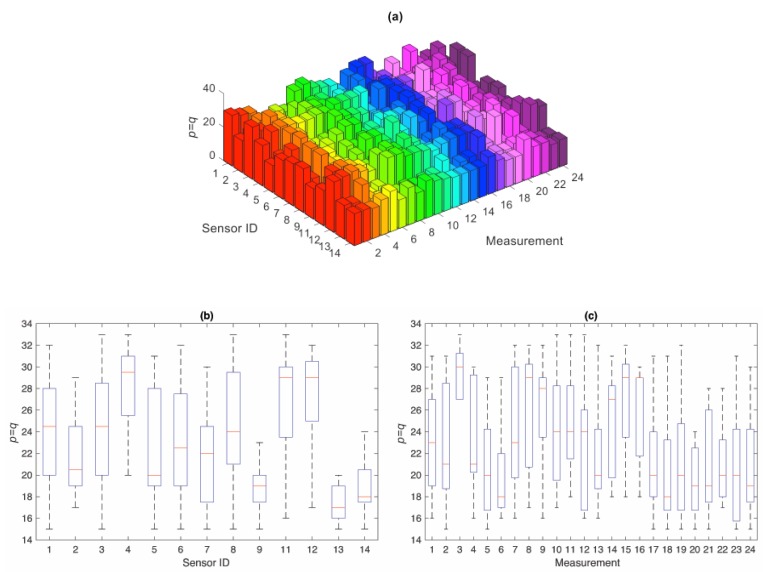
(**a**) Estimated orders *p* = *q* of the ARMA model at each sensor location for the normal condition; box plots of the estimated orders as a function (**b**) of sensor position and (**c**) measurement.

Since with both the CBFE and RBFE algorithms, it is necessary to account for the model orders of each undamaged condition in the current state (Step 4 of Table 1), to avoid handling a large volume of damage-sensitive features and therefore save time and storage space, a model order reduction strategy has been sought. The ARMA orders relevant to all eight days of the baseline have been averaged for each sensor, to move from the formerly mentioned 192 order values to the resulting 24 only. In this way, the average model orders at each sensor location can be used for each day of the normal condition, with no further time-consuming switching or tuning. Figure 2 shows a general view of the obtained average values of *p* and *q* at each sensor location and for each test measurement; further to that, and even though an analysis of the correlation between the model orders and sensor placement, so local features of the structural response, or unknown environmental/loading conditions is beyond the scope of this work, the figure also provides some data concerning the variability of *p* = *q*. Next, the maximum value of the orders has been chosen for the modeling of the vibration responses for all 13 sensors in the undamaged and current states. According to the results gathered in Figure 2, the maximum order turns out to be equal to 33. Since this order enables all the ARMA models to generate uncorrelated residuals, its use assures appropriate response modeling and feature extraction. By using instead any smaller order, for which the model residuals become correlated, the ARMA representations cannot appropriately model the vibration responses and consequently lead to a poor feature extraction.

The prediction error technique proposed in [41] has been adopted to set the AR and MA coefficients of the ARMA(33,33) model for the normal conditions (Step 2 in Table 1). The AR coefficients at each sensor location are finally used as damage-sensitive features of the structure, according to Step 3a in Table 1. The main criterion for assessing the accuracy of a model is through its model residuals [40]; if underfitting does not occur, the orders enable the generation of the sought uncorrelated residuals (see Step 3b in Table 1).

**Figure 3:**
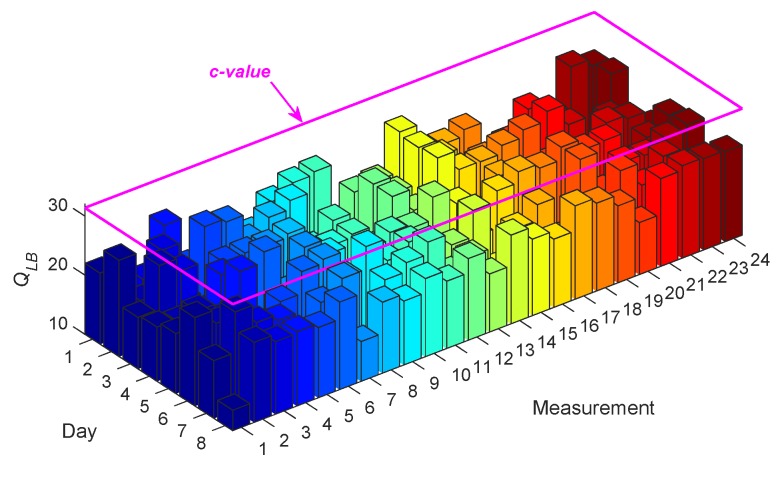
Ljung–Box Q-test (LBQ) test statistics for ARMA(33,33) to model the vibration responses at Sensor 8 for all test measurements on Days 1–8.

The process of accuracy checking has been carried out by a correlation analysis through the LBQ test. Under a significance level (e.g., 5%), the test does not reject the null hypothesis (i.e., the uncorrelatedness of the model residuals) if and only if the test statistic (*Q_LB_*) is smaller than the *c*-value. For example, Figure 3 shows the values of *Q_LB_* obtained with the residuals of ARMA(33,33) at Sensor 8, for all the measurements relevant to the normal conditions; in this case, the *c*-value under the 5% significance level is identical to 31.4101. As can be seen, all test statistics are smaller than the *c*-value, implying the uncorrelatedness of the model residuals and the accuracy of ARMA(33,33) for modeling the vibration responses of Sensor 8 for all measurements on Days 1–8. Note that the same conclusion has been arrived at for all the other sensors.

For modeling the vibration responses in the current state, according to Step 4 in Table 1, the same orders are employed, to set the new model coefficients with the CBFE algorithm (Step 5a) or to use the same ones adopted for the baseline with the RBFE algorithm (Step 5b). Finally, the new damage-sensitive features are estimated in Step 6. By collecting the AR coefficients for all sensor locations and test measurements, the feature matrices result to consist of 10,296 samples (as r=33×13×24=10,296); the matrices **X** and **Z** are, therefore, of sizes 10296 × 8 and 10296 × 1, with the number of columns referring to the undamaged and damaged states being *n* = 8 and *m* = 1, respectively. 

### 4.3. Early Damage Detection

**Figure 4:**
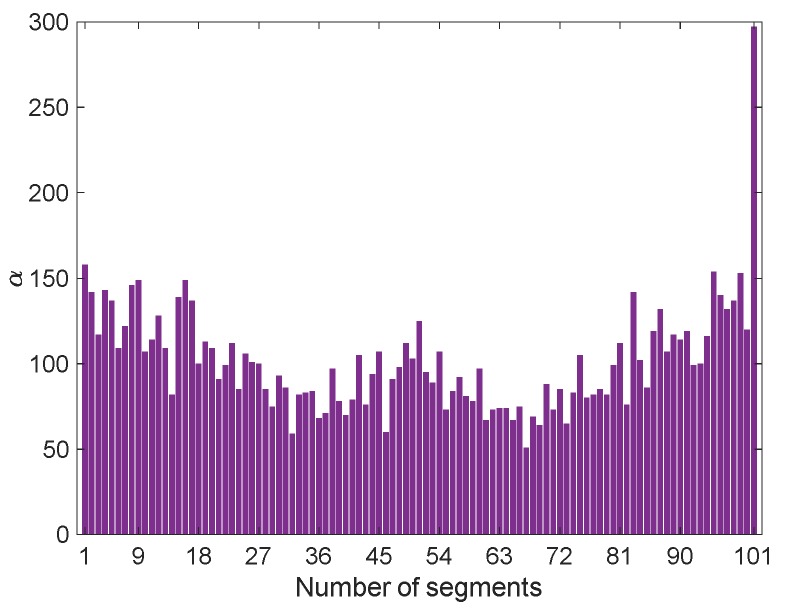
Exemplary segmentation of **x_5_**.

To obtain the vectors **P_xx_** and **P_xz_** through Equations (7) and (8), the arranged feature vector of the damaged state **z***_j_* has been segmented according to the maximum entropy technique (see Section 3.2.). Since this vector consists of 10,296 samples, the adopted PKLD is characterized by *β* = 101, *c* = 101, *β_c_* = 95 and *γ_c_* = 0.0092. The segmentation process has then been performed for the feature vectors of **X** without any arrangement; Figure 4 shows some exemplary results related to *α_h_*, with *h* = 1,…,100, and *α_c_* of **x_5_**. Concerning the number of samples in each partition, in this specific case it has turned out that *α*_1_ = 158 and *α*_101_ = 297; according to the adopted criterion for the segmenting of the vectors in **X**, it is probable that the number of samples in the last partition (*α_c_*) are (even far) larger or smaller than in the others.

The smallest PKLD value of each feature vector of **X** and **Z** is next computed to provide the vectors **P_xx_** and **P_xz_**, each consisting of eight PKLD entries. The entire procedure reads as follows: the values in **P_xx_** are used in Equation (10) to determine the vector **d_PT_**; the divergence values in it are then handled to define the threshold limit which, under a 5% significance level and 95% confidence, amounts to 0.4666; the smallest PKLD values in **P_xx_** and **P_xz_** are used in Equation (9) to obtain **d_PN_**, which consists instead of 64 entries. 

**Figure 5:**
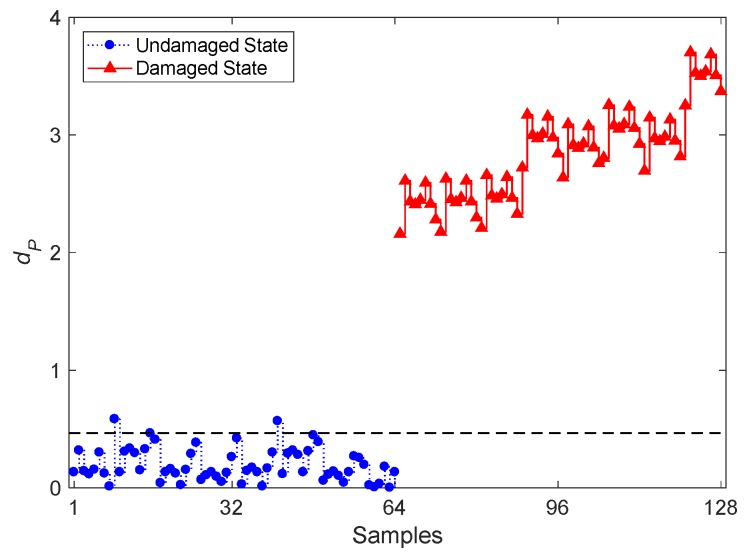
Evolution of the distance value *d_P_* based on the proposed partition-based Kullback–Leibler divergence-nearest neighbor (PKLD-NN) method and on the CBFE algorithm, using the ARMA(33,33) model.

The results in terms of early damage detection by the proposed PKLD-NN method and the CBFE algorithm, on the basis of the ARMA(33,33) model, are reported in Figure 5. In the graph, the first 64 values refer to **d_PT_**, which represents the undamaged state of the bridge; samples 65–128 are instead associated with **d_PN_** and, therefore, with the damaged state. It can be seen that most of the divergence values relevant to the normal condition fall below the threshold limit, with the exception of two samples only. The other way around, all the divergence values related to the damaged state exceed the threshold value to warn about the occurrence of damage. Even without considering the threshold limit, one can clearly distinguish the trend linked to either the normal or the damaged condition. The proposed PKLD-NN method in conjunction with the CBFE algorithm and ARMA modeling, therefore, looks successful in detecting damage, with a remarkable accuracy. These results have been obtained by handling a big data set, with the approach that allowed a reducing of the samples from the initial 898,560,000 acceleration ones to the 128 divergence values specifically designed for damage detection.

### 4.4. Comparative Analyses

In the following, some comparative studies are provided to show the superiority of the proposed methods over state-of-the-art techniques.

**Figure 6:**
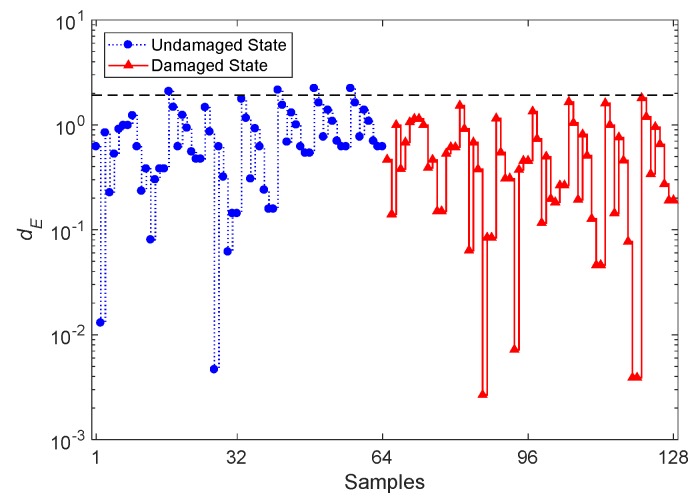
Evolution of the logarithmic distance value *d_E_* based on the classical Euclidean-squared distance-nearest neighbor (ESD-NN) technique and on the CBFE algorithm, using the ARMA(33,33) model.

Since the PKLD-NN method represents an improvement over the ESD-NN one, a comparison between their performances is shown first. With the ESD-NN approach, the process of damage detection departs from the same feature datasets to determine **d_ET_** and **d_EN_**, each collecting 64 divergence values. Figure 6 shows the result of damage detection via the ESD-NN technique and the CBFE algorithm, using the ARMA(33,33) model; the dashed horizontal line is still the threshold value related to the 95% confidence interval of the divergence quantities in **d_ET_**. From the graph, one can discern that all but four divergence values do not exceed the threshold limit, thus leading to a lack of capability to detect damage in the bridge. As all the divergence values for the samples 65–128 fall below the threshold value, the rate of Type II error amounts to an unbearable 100%. On the contrary, with the PKLD-NN method, and regardless of the threshold limit, a poor damage detectability is reported, with divergence values approximately similar for both the undamaged and damaged states. 

**Figure 7:**
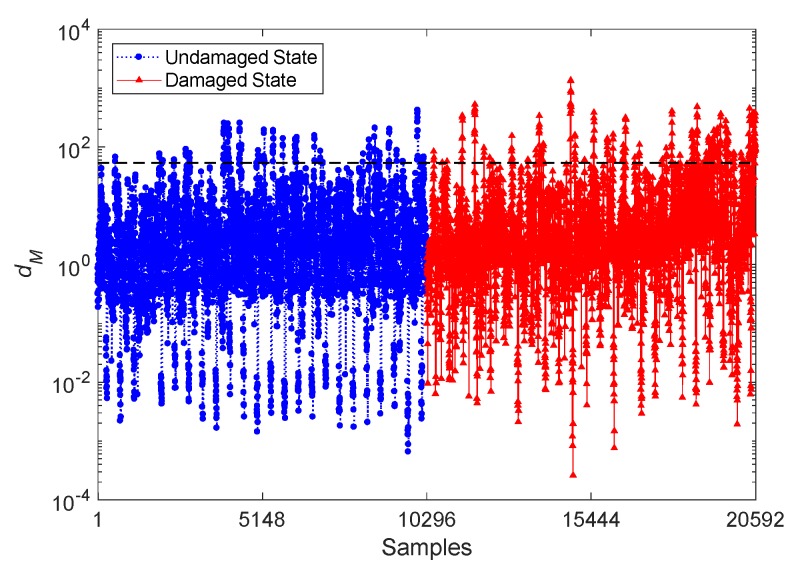
Evolution of the distance value *d_M_* obtained by the conventional Mahalanobis-squared distance (MSD) technique and by the CBFE algorithm, using the ARMA(33,33) model.

If damage detection is performed with the conventional Mahalanobis-squared distance (MSD) technique, which is considered as a popular distance metric in SHM [22,25,37], the feature matrices **X** and **Z** are directly handled to compute the distance values. Figure 7 reports the results relevant to damage detection, where the quantities related to the samples 1–10,296 belong to the undamaged state and the subsequent samples 10,297–20,592 are instead associated with the damaged state. The dashed horizontal line still represents the threshold limit from the 95% confidence interval of the MSD values of the undamaged condition. It clearly emerges that, on average, the MSD quantities for the undamaged state do not exceed the threshold limit; however, several Type II errors show up for the damaged state. Without a threshold limit, discriminating the damaged state from the undamaged one looks difficult, if not impossible. Therefore, even the MSD technique does not prove effective enough for damage detection, due to the poor detectability in the high-dimensional feature sets. 

Table 2 lists the statistics of Type I and Type II errors in damage detection, along with the misclassification rates, via the PKLD-NN, ESD-NN and MSD methods. Values clearly show that the proposed PKLD-NN method provides the best performance, while both the ESD-NN and MSD techniques lead to false negatives with an extremely large percentage of Type II errors. The proposed PKLD-NN method thus outperforms the classical approaches and enables a higher damage detectability.

**Figure 8:**
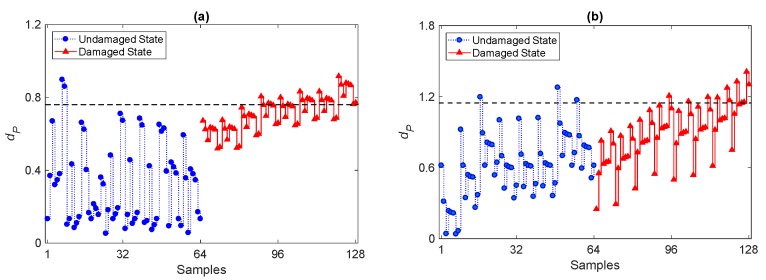
Evolution of the distance value *d_P_* by the proposed PKLD-NN method and by the CBFE algorithm, using models (**a**) ARMA(15,15) and (**b**) ARMA(5,5).

Although the results confirm that the PKLD-NN method is an effective tool for damage detection, the effect of ARMA modeling on the performance of damage detection has not been considered yet. Another analysis is now discussed to show how an inappropriate choice of the orders of the ARMA representation can spoil the performance of PKLD-NN. To this aim, two further model orders have been selected: a first one is linked to the minimum value of the average orders on Days 1–8, which leads to ARMA(15,15); a second one is arbitrarily chosen smaller than the minimum, in order to plug-in the issue of underfitting, and leads to ARMA(5,5). The results are reported in Figure 8 in terms of the time evolution of the *d_P_* distance value via the PKLD-NN method in conjunction with the use of damage-sensitive features extracted from the ARMA(15,15) and ARMA(5,5) models. It can be observed that the method fails in accurately detecting damage in both cases, due to the insufficient orders to extract the sought damage-sensitive features. In the graphs, the majority of the divergence values for samples 65–128 related the damaged state fall below the threshold limit and provide a considerable amount of Type II errors. Hence, the use of features obtained from models ARMA(15,15) and ARMA(5,5) reduces, if not completely spoils the damage detectability of the proposed method. Without a threshold, with ARMA(5,5) it becomes hard to discriminate the damaged and normal conditions; in contrast, with ARMA(15,15), results are not as good as those obtained with ARMA(33,33), but some capability to distinguish the damaged and undamaged conditions is preserved. Although it looks obvious that, by reducing the orders of the ARMA model, the capability to detect damage is progressively decreased, these results allow to quantify how the performance of the method is spoiled; that is, by lowering the orders *p = q*, errors in damage detection first appear and then next, a clear distinction between the undamaged and damaged states is basically lost.

**Figure 9:**
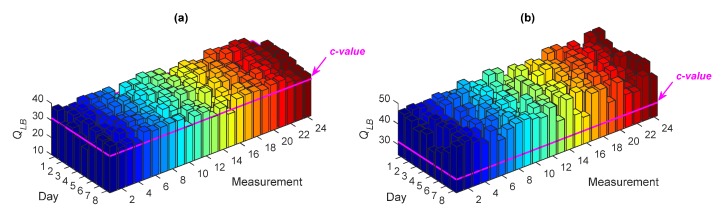
LQB test statistics for (**a**) ARMA(15,15) and (**b**) ARMA(5,5) to model the vibration responses at Sensor 8 for all test measurements on Days 1–8.

The overall results of the comparative analysis are reported in Table 3, in terms of the percentages of Type I and Type II errors, and the misclassification rates for the different ARMA models. It emerges that the use of ARMA(15,15) and ARMA(5,5) causes larger Type II and misclassification errors, to confirm the negative effect of using improper model orders on damage detectability by the proposed PKLD-NN method. In order to also assess the performance of these models from a statistical viewpoint, Figure 9 shows the values of *Q_LB_* associated with the residuals extracted from ARMA(15,15) and ARMA(5,5), at Sensor 8 for all measurements on Days 1–8. The majority of the *Q_LB_* values in Figure 9a and all the values in Figure 9b are larger than the *c*-value; this proves, again, the inaccuracy of the ARMA(15,15) and ARMA(5,5) models.

A final comparison pertains to the assessment of the CBFE and RBFE algorithms. Moving to the efficiency of the considered algorithms in the presence of big data to process, a comparison is provided in Table 4 in terms of the number of features extracted, which are used in the process of damage detection, and the computing time for feature extraction, which are the AR coefficients for the CBFE algorithm and the ARMA residuals for the RBFE algorithm. These data refer to analyses carried out with a computer featuring an Intel Core i7-3770, 3.40–3.90 GHz CPU and 16GB RAM, and reveal that the CBFE algorithm is much more efficient than the RBFE one. To catch the reasons leading to the different computational efforts of the two algorithms, we recall that, besides the algorithmic details pertinent to the two procedures, with the CBFE approach model, coefficients must be tuned both in the undamaged and the damaged states, while with the RBFE approach, this step is carried out just once in the undamaged initial state. Since the dimension of residual datasets extracted by the RBFE algorithm is also equal to the acceleration responses, a huge amount of the samples has to be processed as damage-sensitive features. In other words, the great limitation of the model residuals is that they have the same size of the actual vibration data; if the residuals of the ARMA model are chosen as damage-sensitive features, it is necessary to process the entirety of the 1,010,880,000 data samples. In such a case, the issues linked with big data are still unsolved. The comparison between the two algorithms in terms of computational costs thus proves that the CBFE approach needs a shorter time, and so it is also convenient as far as the time required for data processing is concerned. Overall, the CBFE algorithm is more efficient than the RBFE one in addressing the problem of big data.

As far as the processing issues linked to big data are concerned, the PKLD-NN method is able to provide low-dimensional outputs. In the case study investigated here, moving from the 92,664 feature samples represented by the AR coefficients of the ARMA models, damage detection is tackled by processing only 128 divergence values, as shown in Figure 5. Hence, from the initial 1,010,880,000 samples representing a large volume of vibration measurements, the procedure condensed the necessary information into 92,664 feature samples and finally into 128 distance quantities only.

## 5. Conclusions

In this work, we have discussed an approach to efficiently deal with big data in the process of structural health monitoring of civil structures, based on the statistical pattern recognition paradigm. Feature extraction has been carried out by means of autoregressive moving average modeling. An innovative hybrid divergence-based method, termed partition-based Kullback–Leibler divergence-nearest neighbor (PKLD-NN), has been proposed to detect damage. With the proposed method, a segmentation strategy based on the maximum entropy has been used to partition the feature samples relevant to the undamaged and current states, to be later used for distance calculations. The PKLD method has been shown to improve the conventional Kullback–Leibler Divergence (KLD) method in measuring the discrepancies between two sets of random high-dimensional time series, so it copes with the limitations enforced by big data. The high-dimensional experimental datasets relevant to the Tianjin Yonghe Bridge have been exploited to verify the efficiency and effectiveness of the proposed method, also in comparison with the alternative methodologies available in the literature.

The results have proved that the proposed PKLD-NN method succeeds in detecting damage in the presence of big data. The comparative analyses have revealed that the method is superior to the classical Euclidean-squared distance-nearest neighbor and Mahalanobis-squared distance techniques, leading to a higher damage detectability and yielding a smaller amount of Type I and Type II errors. The comparison between the coefficient-based and residual-based algorithms for feature extraction has also shown that the former is more efficient to cope with large datasets, e.g., in terms of the number of feature samples and computational time.

In this work, the occurrence of damage has been assessed by comparing the distance values with a threshold limit obtained from the standard confidence interval analysis. For future works, it is intended to develop a new threshold estimation technique, especially for small data samples. The fundamental principle of the proposed PKLD-NN method has been based on a distance calculation for the univariate samples, without considering the correlation among them. Therefore, multivariate versions of KLD for a distance calculation are to be considered next.

## Figures and Tables

**Table 1:** Steps of the coefficient-based feature extraction (CBFE) and residual-based feature extraction (RBFE) algorithms based on autoregressive moving average (ARMA) modeling.

Step No.	Feature Extraction Algorithms		State
	CBFE	RBFE	
1	Determine the orders for each vibration response		Undamaged
2	Estimate the model coefficients		
3	(a) Extract the AR coefficients	(b) Extract the ARMA residuals	
4	Use the ARMA orders obtained in Step 1		Current
5	(a) Estimate the new model coefficients	(b) Use the model coefficients obtained in Step 2	
6	(a) Extract the new AR coefficients	(b) Extract the new ARMA residuals	

**Table 2:** Statistics of Type I and Type II errors, and misclassification rates by the PKLD-NN, ESD-NN and MSD methods.

Method	Type I	Type II	Misclassification
PKLD-NN	3.12%	0%	1.56%
ESD-NN	6.25%	100%	53.12%
MSD	3.30%	74.55%	38.92%

**Table 3:** Statistics of Type I and Type II errors, and misclassification rates by the PKLD-NN method, at varying orders *p = q* of the ARMA model.

Model	Type I	Type II	Misclassification
ARMA(33,33)	3.12%	0%	1.56%
ARMA(15,15)	3.12%	64.06%	33.59%
ARMA(5,5)	4.68%	84.37%	44.53%

**Table 4:** Comparison of the performances of CBFE and RBFE algorithms.

Method	Number of Features	Computational Time (Min)
CBFE	92,664	39
RBFE	1,010,880,000	467
